# Increased Plasma Cell-Free DNA Level during HTNV Infection: Correlation with Disease Severity and Virus Load

**DOI:** 10.3390/v6072723

**Published:** 2014-07-15

**Authors:** Jing Yi, Yun Zhang, Yusi Zhang, Ying Ma, Chunmei Zhang, Qi Li, Bei Liu, Zhijia Liu, Jiayun Liu, Xianqing Zhang, Ran Zhuang, Boquan Jin

**Affiliations:** 1 Department of Immunology, The Fourth Military Medical University, 169 Changle West Rd, Xi’an 710032, China; E-Mails: yeluo@fmmu.edu.cn (J.Y.); immuzy@fmmu.edu.cn (Y.Z.); zhangyusi_scu@163.com (Y.Z.); merry_bg20@163.com (Y.M.); hzcm1981@126.com (C.Z.); lq2008@fmmu.edu.cn (Q.L.); liubei83@163.com (B.L.); liuzhijia007@gmail.com (Z.L.); immu_jin@fmmu.edu.cn (B.J.); 2 Department of Blood Transfusion, Xijing Hospital, The Fourth Military Medical University, 15 Changle West Rd, Xi’an 710032, China; E-Mail: zhangxq@fmmu.edu.cn; 3 Department of Clinical Laboratory, Xijing Hospital, The Fourth Military Medical University, 15 Changle West Rd, Xi’an 710032, China; E-Mail: jiayun@fmmu.edu.cn

**Keywords:** cf-DNA, HFRS, Hantaan virus, disease severity

## Abstract

Cell-free DNA (cf-DNA) in blood represents a promising DNA damage response triggered by virus infection or trauma, tumor, *etc.* Hantavirus primarily causes two diseases: haemorrhagic fever with renal syndrome (HFRS) and Hantavirus cardiopulmonary syndrome (HCPS), depending on different Hantavirus species. The aim of this study was to evaluate plasma cf-DNA levels in acute phase of HFRS, and to correlate plasma cf-DNA with disease severity and plasma Hanttan virus (HTNV) load. We observed the appearance of cf-DNA in 166 plasma samples from 76 HFRS patients: the plasma cf-DNA levels peaked at the hypotensive stage of HFRS, and then decreased gradually. Until the diuretic stage, there was no significant difference in plasma cf-DNA level between patients and the healthy control. Exclusively in the febrile/hypotensive stage, the plasma cf-DNA levels of severe/critical patients were higher than those of the mild/moderate group. Moreover, the plasma cf-DNA value in the early stage of HFRS was correlated with HTNV load and disease severity. In most of the patients, plasma cf-DNA displayed a low-molecular weight appearance, corresponding to the size of apoptotic DNA. In conclusion, the plasma cf-DNA levels were dynamically elevated during HFRS, and correlated with disease severity, which suggests that plasma cf-DNA may be a potential biomarker for the pathogenesis and prognosis of HFRS.

## 1. Introduction

Hantaviruses, classified in the *Hantavirus* genus in the family of *Bunyaviridae* [1], are etiological agents of hemorrhagic fever with renal syndrome (HFRS) in the area known as Eurasia, while in the Americas, they manifest as a newly recognized Hantavirus cardiopulmonary syndrome (HCPS). Currently, over 40 hantaviral genotypes have been described, and nearly half of them are pathogenic for humans. In China, generally only Hantaan, Seoul and Puumala viruses are reported to cause severe, common and mild HFRS respectively. HRFS clinical symptoms mainly include thrombocytopenia and, in severe cases, hemorrhage and capillary leakage. The pathogenesis of HFRS has been documented to some extent [2], but is still considerably far from being completely understood. Among all the well-known mechanisms underlying HFRS pathogenesis, immune response may play key roles, involving immune complexes, complement activation, B cell response, T cell response, and HTNV-induced cytokine production, which cause an increase of vascular permeability and tissue injury.

Circulating cell-free DNA (cf-DNA) is detectable DNA fragments in plasma or serum of healthy individuals or patients, probably through cellular apoptosis and/or necrosis [3]. Increased cf-DNA level has been investigated and documented previously in various acute and chronic clinical disorders, including pathogenic microbe infection diseases, tumors, autoimmune diseases, sepsis, myocardial infarction, pre-eclampsia, stroke, and even trauma and chest pain [4,5,6]. In several of the aforementioned conditions, cf-DNA level also had good diagnostic and prognostic values to predict future outcomes [7,8,9]. For example, the copy number of donor-derived cell-free DNA (dd-cfDNA) in blood correlates with acute rejection (AR) in kidney transplant injury, and can be used as a surrogate marker [10]. Although cf-DNA is also detectable in healthy donor plasma samples, it is elevated significantly and presents a pattern of dynamic change under disease conditions. In addition, cf-DNA release is related to cell apoptosis or necrosis and therefore can partly reflect the degree of cellular damage. Cf-DNA is correlated with other laboratory parameters, and therefore can be considered as a novel biomarker to predict the outcome or severity of disease in some disorders. The present study was designed to evaluate plasma cf-DNA levels in an acute phase of HFRS, and to correlate cf-DNA levels with disease severity and plasma Hanttan virus (HTNV) load.

## 2. Results and Discussion

### 2.1. Study Population Characteristics

A total of 186 plasma samples from 96 subjects, including 76 HFRS patients and 20 healthy volunteers were enrolled in this study. Their characteristics and laboratory parameters are summarized in Table 1.

**Table 1:** Demographic and clinical characteristics of 166 plasma samples from 76 haemorrhagic fever with renal syndrome (HFRS) patients.

Characteristic	Characteristic	Value
Disease severity (No. of patients)	Mild	10
	Moderate	22
	Severe	24
	Critical	20
Stage of collection (No. of samples)	Febrile	36
	Hypotensive	9
	Oliguric	40
	Diuretic	56
	Convalescent	25
Age (ys)	Range	9–73
	Mean	42
Sex (No. of patients)	Male	57
	Female	19
Platelets min (10^9^/L)	Range	1–161
	Mean	33.78
Creatinine max (µmol/L)	Range	83.9–931.8
	Mean	403.06
WBC max (10^9^/L)	Range	5.03–91
	Mean	23.82

### 2.2. Cf-DNA Level in HFRS Patients

**Figure 1:**
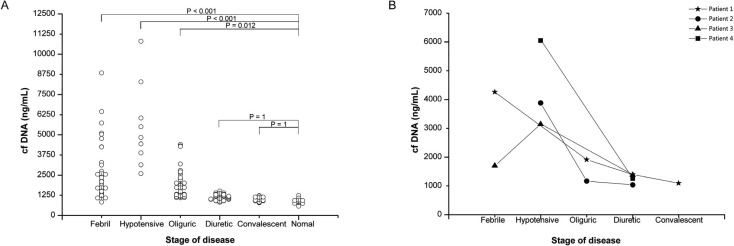
Plasma cf-DNA level in HFRS patients. (**A**) Data were obtained from 166 samples of HRFS patients and 20 samples from healthy controls; (**B**) cf-DNA level in serial samples from four patients who had samples for two, three or four stages. *p* values of less than 0.05 were considered statistically significant.

cf-DNA was detected in all the samples, ranging from 576 ng/mL to 10,800 ng/mL. As shown in Figure 1, the mean values of cf-DNA at different stages showed a dynamic increase and gradual decrease pattern, at serial febrile, hypotensive, oliguric, diuretic, and convalescent stages. The plasma cf-DNA values taken at the febrile stage were significantly higher (2684.22 ng/mL, 3.1 fold) than the control values (861.48 ng/mL) taken from healthy volunteers (*p* ˂ 0.001, Figure 1A). The plasma cf-DNA level reached a peak point in the hypotensive stage (5503.33 ng/mL, 6.4 fold than Ctrl), and remained significantly higher than the control (*p* = 0.012, Figure 1A) before declining in the oliguric stage (1909.34 ng/mL, 2.2 fold than Ctrl). The plasma cf-DNA level during the diuretic (1119.71 ng/mL, 1.3 fold than Ctrl) and convalescent stages (1004.16 ng/mL, 1.2 fold than Ctrl) were close to the normal levels from healthy volunteers (*p* = 1, *p* = 1, Figure 1A). The dynamic change of plasma cf-DNA in four representative patients who had serial samples from two to four stages during the course of the disease also showed that cf-DNA values reached the peak point in the hypotensive stage and gradually declined and decreased to a normal level with the progress of the disease (Figure 1B). 

### 2.3. Cf-DNA Level and Disease Severity of HFRS

**Figure 2:**
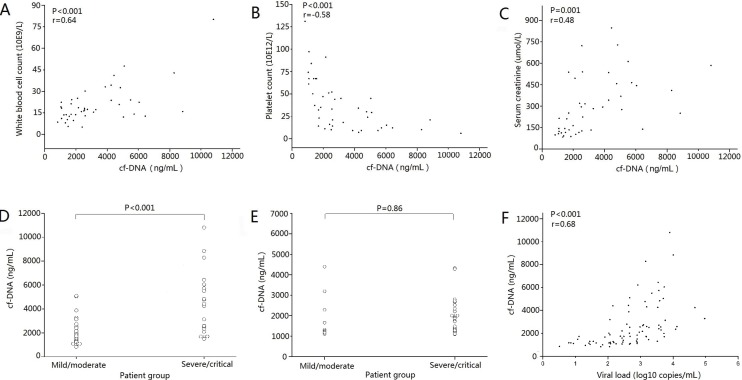
Relationship between plasma cf-DNA level in acute phase (febrile/hypotensive stages) from 44 samples and the peak value of white blood cell count (**A**) and the lowest value of platelet count (**B**), and the peak value of serum creatinine (**C**). Comparison of plasma cf-DNA level between the mild/moderate group and the severe/critical group in febrile/hypotensive stage (**D**) and oliguric stage (**E**). Positive correlation of plasma cf-DNA level with viral load in febrile/hypotensive stage (**F**). *p* values of <0.05 were considered statistically significant.

We analyzed the relationship between plasma cf-DNA values in febrile/hypotensive stages and three laboratory serological parameters that could represent the severity of the disease. The plasma cf-DNA levels positively correlated with maximum white blood cell count (r = 0.64, *p* ˂ 0.001, Figure 2A) and maximum serum creatinine (r = 0.48, *p* = 0.001, Figure 2C), and negatively correlated with minimum platelet count (*p* ˂ 0.001, R = −0.58, Figure 2B). Subsequently, the relationships between disease severity and cf-DNA values in plasma samples collected in the febrile/hypotensive stage and the oliguric stage were analyzed, respectively. The mean value of plasma cf-DNA from the febrile/hypotensive stage in the severe/critical group was significantly higher than that in the mild/moderate group (4406.81 ng/mL *vs.* 2139.65 ng/mL; *p* ˂ 0.001, Figure 2D). However, no significant difference was found between the severe/critical group and the mild/moderate group (1894.31 ng/mL *vs.* 1957.78 ng/mL; *p* = 0.86, Figure 2E) in the oliguric stage. We also analyzed the relationship between plasma cf-DNA values in febrile/hypotensive stages and the viral load, which was reported to correlate positively with disease severity [2], and found that there is a significant positive correlation of plasma cf-DNA levels with viral load (*p* ˂ 0.001, R = 0.68, Figure 2F).

### 2.4. Qualitative Analysis of Plasma cf-DNA

**Figure 3:**
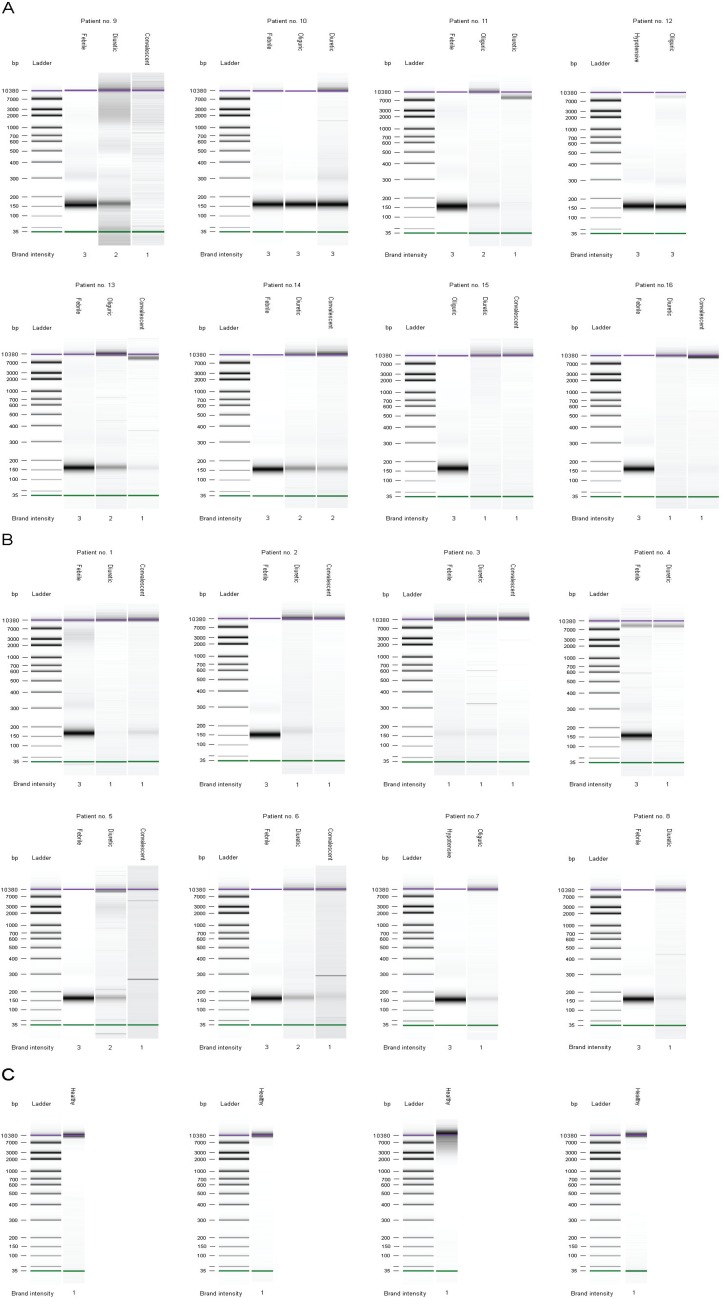
Qualitative analysis of plasma cf-DNA in eight severe/critical patients (**A**); eight mild/moderate patients (**B**); and four healthy controls (**C**) after NucleoSpin Plasma XS kit extraction. The lower, thin solid lines indicate the low weight (35 bp) DNA marker and the upper thick solid lines indicate the high weight (10,380 bp) DNA marker.

Qualitative analysis of plasma cf-DNA revealed that in most patients cf-DNA displayed a low-molecular weight appearance, corresponding to the size of apoptotic DNA fragments (150–200 bp) (Figure 3A,B). The visually graded maximum cf-DNA band intensity correlated positively with the maximum quantity of total plasma cf-DNA (r = 0.46, *p* = 0.002). In most of the plasma samples taken from convalescent stages, the low-molecular weight cf-DNA band was completely absent (Figure 3A,B). In most severe/critical patients, the low-molecular weight bands were still visible during the diuretic stage (Figure 3A), whereas in most mild/moderate patients the low-molecular weight bands were visible during the febrile, hypotensive and oliguric stages and completely absent or markedly weakened during the diuretic stage (Figure 3B). It is noteworthy that in one mild patient the low-molecular weight band was markedly weakened even during the febrile stage (Figure 3B). In the qualitative analysis of healthy controls’ sera samples in contrast to patients’ sera, no distinguishable low-molecular weight (150–200 bp) patterns of cf-DNA were detected (Figure 3C).

### 2.5. Cf-DNA Sequencing and Alignment

The cf-DNA extracted from 10 HFRS patients’ sera samples were sequenced using the Illumina Hiseq platform. A paired-end library (100 bp) was constructed for cf-DNA sequence analysis. In total, we generated 1.348 Gb clean data and 12,940,782 of usable sequences reads for the sera cf-DNA. After alignment, 99.86% of useful reads mapped at human genome sequences (12,923,157/12,940,782 reads, Ref. Homo_sapiens.GRCh37.75). After alignment to the HTNV virus genome, we found that the remaining 14,676 reads, 0.14% of all the useful reads, which unmapped at the human genome, did not map at HTNV genome either. 

### 2.6. Discussion

The maximum plasma cf-DNA levels during the acute phase of HFRS were significantly higher than those from the healthy control samples. The maximum plasma cf-DNA levels were correlated positively with maximum white blood cell count, serum creatinine and inversely correlated with blood platelet count. The visually graded maximum cf-DNA band intensity was correlated positively with the maximum quantity of total plasma cf-DNA. In Hantavirus infected diseases, cf-DNA could been recognized as a novel biomarker for disease severity, similar to interleukin-6, pentraxin-3, C-reactive protein, indoleamine 2,3-dioxygenase, soluble urokinase-type plasminogen activator, GATA-3 and Mac-2 binding protein [11]. In this study, we found that plasma cf-DNA level was correlated with HTNV viral load and some other laboratory parameters, which reflect the severity of HFRS. We analyzed the relationship between plasma cf-DNA values in febrile/hypotensive stages and three laboratory serological parameters that could represent the severity of the disease, including maximum white blood cell count, maximum serum creatinine and minimum platelet count. The maximum level of white blood count and maximum serum creatinine were strongly positively correlated with the severity of HFRS. Moreover, the platelet count showed a significant negative correlation with disease severity. The results showed that in HFRS patients, there was a significant positive correlation between plasma cf-DNA and the severity of disease. 

Qualitative analysis of plasma cf-DNA revealed that in almost all the patients, plasma cf-DNA displayed a low-molecular weight appearance, corresponding to the size of apoptotic DNA (150–200 bp). In most clinical disorders, such as trauma, shock, infection and heart failure and so on, DNA fragments released from cells undergoing programmed cell death or acute cellular injury are the main sources of disease-associated elevation of cf-DNA [12]. Otherwise, in tumors, cf-DNA may be primarily released by living cancer cells in addition to apoptotic or necrotic cancer cells, caused by some alternative mechanisms [13]. Cf-DNA, due to its significant increase in some tissue injury, plays an important role in initiating organism response. In patients with severe infection, the innate immune system responds to a set of evolutionarily conserved molecules known as pathogen-associated molecular patterns (PAMPs), which are expressed by a variety of pathogens. In addition to PAMP expressed in invading microorganisms, immune response is also initiated and perpetuated by endogenous native molecules, named damage-associated molecular pattern (DAMP), such as cell-free DNA, high mobility group box-1 (HMGB1) and other alarmins [14,15]. DAMP can act through signaling pathways shared with PAMPs, initiating a similar innate immune response even in the absence of microbial infection. The endogenesis alarmins, released by injury cells, also as a positive feedback mechanism, became one explanation for the cause of acute and strong innate immune response. In addition, IFI16, a nuclear DNA sensor has been reported as circulating protein in blood of patients with autoimmune diseases, which signals tissue damage, suggesting a new pathogenic and alarmin function through which this protein triggers the development of autoimmunity [16]. The cf-DNA released from injury tissue and dead cells can bind Toll like receptor-9 (TLR9), an intracellular receptor that responds to bacterial or viral DNA molecules. Considering its low base level in healthy control and significant high peak at acute phase of the disease, cf-DNA might be able to play an alarmin role in HFRS and initiate immune response. Further investigations are required to determine the mechanisms by which cf-DNA triggers innate immune response during acute infection, and the virus-host interactions, in order to gain a better understanding of the change of cf-DNA levels.

## 3. Experimental Section

### 3.1. Subjects

One hundred and eighty six archived plasma samples previously collected from 76 patients with HFRS during 2009–2011 and from 20 healthy volunteers were analyzed. Plasmas were stored at −80 °C until being used. This study was approved through the Institutional Review Board of the Fourth Military Medical University, and written informed consent was provided by each individual.

### 3.2. Clinical Data and Laboratory Tests

Clinical data and laboratory findings were registered on admission. The patients were categorized as severe-to-critical group and mild-to-moderate group based on the clinical and laboratory parameters used in the diagnostic criteria for HFRS in China [17]. Laboratory tests included blood platelets (×10^9^/L), serum creatinine level (μmol/L) and white blood cell count (×10^9^/L). The viral load data were also available from the study we had recently performed [2]. 

### 3.3. Quantification of Total Plasma cf-DNA

The cf-DNA analysis was performed afterwards from frozen samples stored at −80 °C. The amount of total cf-DNA was determined directly in plasma, using the Quant-iT^TM^ dsDNA High-Sensitivity Assay Kit and a Qubit^TM^ fluorometer (Invitrogen, Carlsbad, CA, USA) according to the manufacturer’s instructions and previous study [18]. Plasma samples were analyzed in duplicate and the mean of the two values was used as the final value.

### 3.4. Extraction and Qualitative Analysis of Plasma cf-DNA

Qualitative analysis of cf-DNA was performed in 16 randomly selected patients’ cases (severe-to-critical group and mild-to-moderate group, n = 8, respectively) and 4 healthy controls. Plasma cf-DNA was extracted using the NucleoSpinH Plasma XS Kit (MACHEREY-NAGEL GmbH & Co., Duren, Germany), which is designed for isolation of low-molecular-weight cf-DNA (50–1000 bp). Extracted cf-DNA samples were stored at −80 °C until further analyses were performed. Extracted cf-DNA samples were analyzed with the Quant-iT^TM^ dsDNA High-Sensitivity Assay Kit and an Agilent 2100 Bioanalyzer equipped with Expert 2100 software [18] according to the manufacturer’s instructions (Agilent Technologies Inc., Santa Clara, CA, USA) and previous study [19]. For each sample, the appearance and intensity of low-molecular-weight cf-DNA was estimated visually and graded as follows: 1 = no visible cf-DNA or weak intensity, 2 = intermediate intensity, 3 = strong intensity.

### 3.5. Illumina Sequencing

For sequencing, 10 HFRS patients’ sera samples were randomly selected, and the cf-DNA extractions were sequenced using the Illumina Hiseq2000 platform (Shanghai Personal Biotechnology Co., Ltd., Shanghai, China). A paired-end library (100 bp, costumed genomic library and sequenced using paired-end reads) was constructed for cf-DNA sequence analysis. Library preparation and sequencing followed the manufacturer’s instructions, and sequence reads were collected from the Illumina data processing pipeline.

Data filtering. The following types of reads were filtered out: (1) adapter sequence; (2) final sequence shorter than 50 bp; (3) reads with average quality below Q20; and (4) reads with unidentified nucleotides.

Genome assembly. The cf-DNA with short reads was assembled to human genome sequences by BWA software [20,21,22]. Human genome sequence (Homo_sapiens.GRCh37.75.dna.toplevel.fa) [23] and HTNV genome sequences (GenBank: X55901.1, M14626.1, M14627.1) were used for reference [24].

### 3.6. Statistical Analysis

Statistical analysis was performed by statistical software SPSS Version 12.0 [25] and refers to previous publications [19,26]. For describing the data, medians and ranges were given for continuous variables. The Skewness and Kurtosis test was used for testing the normal distribution of continuous variables. For group comparison, Mann-Whitney tests were used for continuous variables which were not normally distributed. Wilcoxon’s test was used to compare two related samples which were not normally distributed. The relationship between cf-DNA and the clinical laboratory parameters was evaluated using the Spearman rank correlation test. All tests are two-sided, and statistically significant *p*-values are given. *p* values of <0.05 were considered statistically significant and bonferroni correction was used when necessary.

## 4. Conclusions

Circulating cf-DNA levels in plasma from HFRS patients were elevated in the early stages during HTNV infection, and gradually decreased to a normal level in the diuretic stage. In the early stages of HFRS, patients in the severe/critical group were found to have a higher plasma cf-DNA level than those in the mild/moderate group, and plasma cf-DNA levels in the febrile/hypotensive stages were positively correlated with the peak value of serum creatinine and white cell count, and negatively correlated with the lowest value of the platelet count, suggesting an association between cf-DNA level and disease severity of HTNV infection. In addition, the plasma cf-DNA levels were correlated with the apoptotic cf-DNA-band intensity. A significant positive correlation between cf-DNA and HTNV viral load in plasma indicated that cellular apoptosis or necrosis induced by HTNV infection might be involved in the pathogenesis of HFRS. 

In conclusion, this is the first study to describe the plasma circulating cf-DNA levels in HFRS patients and determine the correlation between plasma cf-DNA level and HTNV virus load, disease course and severity. We found that the viral load and cf-DNA in plasma from HFRS patients were both positively correlated with disease severity, suggesting that plasma cf-DNA level may be used as a prognostic biomarker to monitor and/or predict the severity of clinical manifestations of HFRS. Our findings provide experimental data for a better understanding of the role of HTNV RNA load as well as plasma cf-DNA during HFRS. 
